# Everyday Challenges for Individuals Aging With Vision Impairment: Technology Implications

**DOI:** 10.1093/geront/gnad169

**Published:** 2023-12-20

**Authors:** Elena T Remillard, Lyndsie M Koon, Tracy L Mitzner, Wendy A Rogers

**Affiliations:** Center for Inclusive Design and Innovation, Georgia Institute of Technology, Atlanta, Georgia, USA; Life Span Institute, University of Kansas, Lawrence, Kansas, USA; Person in Design, Atlanta, Georgia, USA; College of Applied Health Sciences, University of Illinois Urbana-Champaign, Champaign, Illinois, USA

**Keywords:** Aging with disability, Blind, Community participation, Instrumental activities of daily living, Low vision

## Abstract

**Background and Objectives:**

There are growing numbers of older adults with long-term vision impairment who are likely to experience everyday activity challenges from their impairment in conjunction with age-related changes. Technology has potential to support activity engagement. To develop effective technologies and interventions, we need to understand the context of activity challenges and identify unmet support needs.

**Research Design and Methods:**

The Aging Concerns, Challenges, and Everyday Solution Strategies (ACCESS) study is a mixed-method approach to explore everyday challenges of people aging with long-term disabilities. Participants included 60 adults aging with long-term vision impairment (63% female; M age = 67, *SD* = 4.6) who completed in-depth, structured interviews exploring the nature of everyday challenges and their unmet support needs for activity engagement. We conducted a content analysis using a deductive and inductive approach to build a detailed coding scheme of challenge codes and subcodes.

**Results:**

The analyses provided detailed insights about the nature of challenges people aging with vision impairment experience when performing specific instrumental activities of daily living (IADLs) in the context of home maintenance, transportation, shopping/finance, and managing health. Vision-related challenges and participation restrictions were identified for several activities that require reading, navigation, and identification (e.g., shopping, medication management, public transportation). Emergent challenge themes for performing IADLs included personal limitations (e.g., physical, cognitive, financial) and environmental barriers (e.g., accessibility, technology, transportation).

**Discussion and Implications:**

Contextual examples of IADL challenges among individuals aging with vision impairment highlight opportunities for technology design and innovation to support participation in everyday activities.

## Background

Vision impairment can create barriers to engaging in everyday activities, which may inhibit one’s independence, community participation, and overall well-being. The term vision impairment is an umbrella term to describe eyesight that cannot be corrected to a normal level, from mild vision impairment to total blindness, due to a variety of eye conditions or diseases (National Academies of Sciences Engineering and Medicine, 2016; World Health Organization, 2019). A recent framework conceptualized how vision impairment can affect older adults’ functional ability in multiple domains, including physical, cognitive, and psycho-social abilities (Swenor et al., 2020). In line with the International Classification of Functioning, Disability and Health (ICF; World Health Organization, 2001), Swenor et al. indicated that the impact of vision impairment on one’s ability to perform daily activities is dependent on several contextual factors, including the characteristics of the vision impairment (e.g., cause, degree, functional limitations), personal factors (e.g., comorbidities), and environment (e.g., social supports). There is heterogeneity among people aging with vision impairment in terms of activity challenges and support needs (Remillard et al., 2020). Technology provides an opportunity to address these challenges, thereby facilitating activity engagement, performance, and independence (Harrington et al., 2015).

The 2019 U.S. Census American Community Survey estimated that 6% of older Americans (over age 65) have a vision impairment, with prevalence increasing to 9% for Americans over age 75 (Erickson et al., 2022). A recent analysis of the 2021 National Health and Aging Trends (NHATS) study, which incorporated objective measures of visual functioning, estimated the prevalence to be higher, with over 1 in 4 U.S. older adults (ages 71+) having vision impairment (Killeen et al., 2023). The number of older Americans with vision impairment is expected to grow with the aging of the U.S. population (Varma et al., 2016). In contrast to younger counterparts, older adults with vision impairment are likely to have a greater number of comorbid health conditions, such as stroke, hypertension, arthritis, and diabetes (Crews et al., 2017; Steinman, 2016). They may also experience a variety of normative age-related declines (e.g., mobility, hearing, cognitive; Czaja et al., 2019). Collectively, these factors can exacerbate functional limitations for older adults with vision impairment (Swenor et al., 2020).

Among this population, a subset of individuals has long-term vision impairment due to vision conditions or eye injuries acquired earlier in life. These individuals are subject to the unique circumstance of managing their long-term vision impairment, as well as age-related conditions, that together can create significant barriers to activity performance and increase risk of disability (Mitzner et al., 2018). There are known socioeconomic disadvantages for people aging with long-term disabilities, including employment and lower income, as well as greater likelihood of poor health behaviors (e.g., sedentary lifestyle; Clarke & Latham, 2014).

Although vision impairment can affect a wide range of activities, certain types of activities tend to be more affected than others. Basic activities of daily living (ADLs; Katz et al., 1970), such as bathing and eating, may be generally less affected by visual impairment, as they are fundamental tasks ingrained in everyday routines and often occur in the familiar home environment, which can facilitate adaptations (Hochberg et al., 2012). In contrast, instrumental activities of daily living (IADLs), which represent key tasks for independent living, are more complex and visually demanding (Berger & Porell, 2008; Lawton & Brody, 1969). Common IADLs include cooking, cleaning, managing finances, managing medications, using the telephone, and transportation (Graf, 2008; Lawton & Brody, 1969). The visual abilities required to perform IADLs vary by specific activity, but include reading, identifying and manipulating items, navigating spaces, using fine motor movements, and lifting objects.

Home-based IADLs, such as housework, meal preparation, medication management, and money management, have been reported as particularly difficult among older adults with vision impairment (Hochberg et al., 2012; Peres et al., 2017; Steinman, 2016). Vision impairment can contribute to mobility challenges among older adults, including limitations with walking, navigation, and physical activity, as well as fear of falling (Bayles et al., 2022; National Academies of Sciences Engineering and Medicine, 2016). Community-based activities that involve outdoor mobility and transportation, such as going to appointments or shopping, have also been documented as difficult activities (Cimarolli et al., 2012; Hochberg et al., 2012). Moreover, given that many individuals with vision impairment do not drive, they often experience issues with transportation availability, accessibility, and affordability (Bleach et al., 2020; Crudden et al., 2015). Research has identified activities that are difficult for people aging with vision impairment, but little is known about the *context* of their challenges.

Functional limitations with everyday activities can lead to a loss of independence and have negative psychological and social health consequences. Indeed, people with vision impairment are at greater risk for depression, anxiety, and poor quality of life (Demmin & Silverstein, 2020; National Academies of Sciences Engineering and Medicine, 2016; Nyman et al., 2012; Renaud & Bédard, 2013). Activity and participation restrictions in the home and community are associated with loneliness and social isolation, which are prevalent among older adults with vision impairment (Brunes et al., 2019; Coyle et al., 2017). Hence, supports are critically needed to assist with IADL engagement, thereby supporting aging-in-place, functional independence, quality of life, and well-being.

Innovative technology solutions have the potential to improve the lives of those aging with long-term vision impairments by providing needed support for daily activities. In the past two decades, there has been rapid evolution and growth in development of assistive technology (AT) for people with vision impairment (Bhowmick & Hazarike, 2017). Assistive technology refers to a variety of supports, including equipment, devices, and systems to help users engage in daily activities, such as housework or shopping. Common examples of vision AT are screen readers, magnifiers, scanners, and navigational canes. Accessibility tools for people with vision loss are also integrated into modern personal computing devices (e.g., smart phones, computers, tablets), such as enlarged text, enhanced contrast, and voice to text. Emerging technologies include robots to support wayfinding (e.g., Liu et al., in press).

Technology supports will only be helpful if they are successfully adopted. However, many assistive technologies are underused by older adults with vision impairment. Technology adoption among older adults with vision loss may be influenced by number of factors including usefulness, ease of use/usability, accessibility, cost, safety, and compatibility with the user’s attitudes, behaviors, and environment (Kim, 2021; McGrath & Corrado, 2019). Perceived usefulness and ease of use are two of the most significant predictors of technology adoption (Mitzner et al., 2016). Hence, to facilitate the use of technology supports developers must design technologies to support unmet needs and research must explore usability challenges of existing technologies.

People aging with long-term vision impairment have been navigating challenges with everyday activities for an extended period, for some, most, or all, of their lives. These individuals offer a unique opportunity to investigate persisting activity challenges (i.e., unmet support needs) as well as technology experiences. The objective of this study was to explore the nature and context of challenges experienced by adults aging with long-term vision impairment when performing IADLs and the specific factors contributing to their challenges. These rich insights can be used to drive the design and development of innovative technologies (Harrington et al., 2015; Mitzner et al., 2018).

## Aging Concerns, Challenges, and Everyday Solution Strategies Study

The Aging Concerns, Challenges, and Everyday Solution Strategies study (hereafter ACCESS) is a mixed-method exploration of everyday challenges of people aging with disabilities (Koon et al., 2020; Remillard et al., 2018). Covering a wide range of everyday activities in the home and community, ACCESS investigated the breadth and depth of activity-specific challenges as well as the strategies and solutions employed to manage the challenges. Participants were 180 adults with long-term disabilities: 60 with mobility impairment, 60 with vision impairment, and 60 who were deaf. The current paper focuses on the vision group.

The interviews provided detailed insights about the challenges people aging with vision impairment experience performing IADLs, which are key to independent living. ACCESS covered a broad range of IADLs, including activities from the original scale developed by Lawton and Brody (1969), as well as from extended scales that capture a broader range of activities (Fieo et al., 2014; LaPlante, 2010). Specific aspects of IADLs were assessed. For example, instead of focusing on the high-level category of “transportation” as an activity in and of itself, participants were asked about a variety of transportation modes (e.g., getting a ride from a friend or family member; using a taxi/Uber/Lyft; flying on an airplane). Similarly, different methods for shopping (i.e., in-person or online) were explored.

The primary research questions were twofold. First, what is the nature of challenges (e.g., type, frequency, context) people aging with vision impairment experience in performing a broad range of IADLs? Second, what are unmet support needs for IADL performance among people aging with vision impairment? The findings provide guidance for technology design and innovation to support activity participation and independence among this population.

## Research Design and Methods

### Participants

ACCESS was conducted at the University of Illinois Urbana-Champaign and the Georgia Institute of Technology with Institutional Review Board approval from each university. Participants were recruited through outreach to local and national organizations for persons who were blind or had visual impairments, through flyer distribution, social media postings, and word-of-mouth referrals. Eligible participants were age 60–79, who self-identified as having a long-term vision impairment (serious difficulty seeing, even when wearing glasses or contact lenses) that began prior to the age of 50, fluent in English, and resided in the United States.

There were 60 participants with vision impairment (M = 67; *SD* = 4.6), 38 females and 22 males. Causes of vision impairment were diverse with the most common being retinal damage/condition (e.g., retinitis pigmentosa, macular degeneration; 40% of sample), followed by congenital condition or abnormality (e.g., morning glory syndrome, congenital rubella syndrome; 26%), and nerve damage/condition (e.g., glaucoma, optic nerve atrophy; 24%). Other causes, each representing 3% of the sample, included: chronic eye inflammation, cataracts, and other eye injury or damage (e.g., gunshot wound, computer vision syndrome). The mean age of onset of vision impairment was 12 years (*SD *= 14.2), ranging from birth to age 49. The mode for age of vision impairment onset was 0 years (i.e., from birth; *n* = 24). For duration of having a vision impairment, the mean was 56 years (*SD* = 15.4), the mode was 65 (*n* = 4), and range was 16 to 76 years.

Table 1 provides information about participants’ socioeconomic and health characteristics. The majority had some college education, were predominately White/Caucasian (59%) and married (42%), with an annual income of less than $25,000 (41%). In addition, the majority rated their health as good (51%) or very good (27%).

**Table 1.** Participant Characteristics

Variable	Categories	*n*	%
Education	< High school	0	0
	High school graduate/GED	14	23.7
	Vocational training	3	5.1
	Some college/associate’s degree	10	16.9
	Bachelor’s degree	14	23.7
	Master’s degree	14	23.7
	Doctorate degree	4	6.8
Race	White/Caucasian	35	59.3
	Black/African American	17	28.8
	Other	5	8.5
	More than one race	1	1.7
	Do not wish to answer	1	1.7
Marital status	Single	10	16.9
	Married	25	42.4
	Separated	2	3.4
	Divorced	12	20.3
	Widowed	9	15.3
	Do not wish to answer	1	1.7
Income	<$25,000	24	40.7
	$25,000–$49,999	15	25.4
	$50,000–$74,999	6	10.2
	>$75,000	8	13.6
	Do not wish to answer	5	8.5
	Do not know for certain	1	1.7
Perceived health	Poor	0	0
	Fair	5	8.5
	Good	30	50.8
	Very good	16	27.1
	Excellent	7	11.9
	Do not wish to answer	1	1.7

*Note:* Data were missing for one participant, so the cells sum to 59.

### Procedure

ACCESS method details are in Remillard et al. (2018) and Koon et al. (2020). To summarize, after telephone screening eligible participants completed two questionnaires (45–60 min) via online survey, mailed paper copies, or phone to assess demographics, health, and vision impairment. Interviews (60–90 min) were conducted by phone or in-person by trained research team members, including: 1 research scientist who is a gerontologist (author), 1 postdoc with a background in sport and exercise science (author), and 5 graduate students with fields of study including: engineering psychology, community health, and biomedical engineering. See technical report for complete interview guide (Remillard et al., 2018). All participants provided verbal informed consent and received $30 compensation. Interviews were audio-recorded and transcribed verbatim.

The structured interviews covered six broad activity categories: Outside the Home; Around the Home; Shopping/Finances; Transportation; Health; and Basic Activities. For each category, participants were asked about 5–8 specific activities that were guided by the literature and findings from subject matter expert interviews (Preusse et al., 2016). Participants rated their difficulty with specific activities (1 = not at all difficult, 2 = a little difficult, 3 = very difficult, or not applicable). For their most difficult activity in each category, participants answered open-ended follow-up questions probing the specific aspect of the activity that created the most challenge for them and how they managed that challenge (e.g., assistance from others, tools or technologies, own methods, or other strategies; Table 2).

**Table 2.** Follow-up Interview Questions for the Activity Identified as ‘Most Difficult’ in Each Category

Questions
1. Thinking about [*insert most difficult activity*], what aspect or part of this creates the most challenges for you?
2. How do you handle this challenge?
3. Do you use any sort of devices, tools, or technologies to help you with this {task/activity}?
4. Do you use any sort of devices, tools, or technologies to help you with this {task/activity}?
5. Do you use any of your own methods or things you came up with to help you do that {task/activity}?
6. Do you get help from anyone (e.g., services, care-providers, family members) to do that {task/activity}?

### Data Analysis

We conducted a content analysis (Erlingsson & Brysiewicz, 2012; Vaismoradi & Snelgrove, 2019; Vaismoradi et al., 2013) with four members of the research team using both deductive (Harrington et al., 2015; Preusse et al., 2016; Remillard et al., 2019; Rogers et al., 1998) and inductive approaches (Elo & Kyngas, 2008). We iteratively developed a coding scheme that included challenge codes, subcodes, definitions, and example participant quotes. We discussed and revised the coding scheme with the entire research team until consensus was reached (see Koon et al., 2019, 2020). Transcript coding was conducted using the qualitative software program MAXQDA. All four researchers coded a sample transcript until independent coding reliability and agreement were met (*r* = 0.85). This process was repeated for four additional sample transcripts to ensure reliability across coders; as needed, the team modified definitions and added examples to improve clarity of the coding scheme. Each transcript (*N* = 60) was coded by one of the four researchers (random assignment). See Supplementary Material for complete coding scheme.

**Figure 1.**
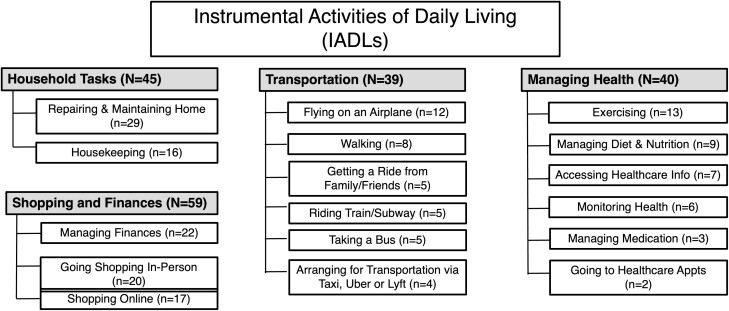
Instrumental activities of daily living (IADL) categories. Some participants reported more than one category as “Most Difficult” yielding 183 total response.

The coding scheme was applied to the units of analysis, defined as participants’ responses to the question, “What aspect or part of this activity creates the most challenge for you?” for the following IADL activity categories: Household Tasks; Shopping and Finances; Transportation; and Health Management (see Figure 1 for activities). The category “Household Tasks” includes IADLs in the “Around the Home” ACCESS study category. The activity of “Driving” was excluded, as most of the participants in the sample did not drive.

We present frequencies of challenge codes reported across the four IADL activities. For each category, we highlight challenges for specific activities and present illustrative participant quotes about the context of these challenges. Quotes are not exhaustive but rather provide rich, descriptive information about activity challenges specific to adults aging with vision impairment.

## Results

### IADL Challenges

Figure 1 shows the distribution of the most difficult IADLs. For the Household Tasks category, the most frequently discussed activity was repairing and maintaining home; for Shopping and Finances, it was managing finances; for Transportation it was flying on an airplane; and for Managing Health it was exercising.

**Figure 2.**
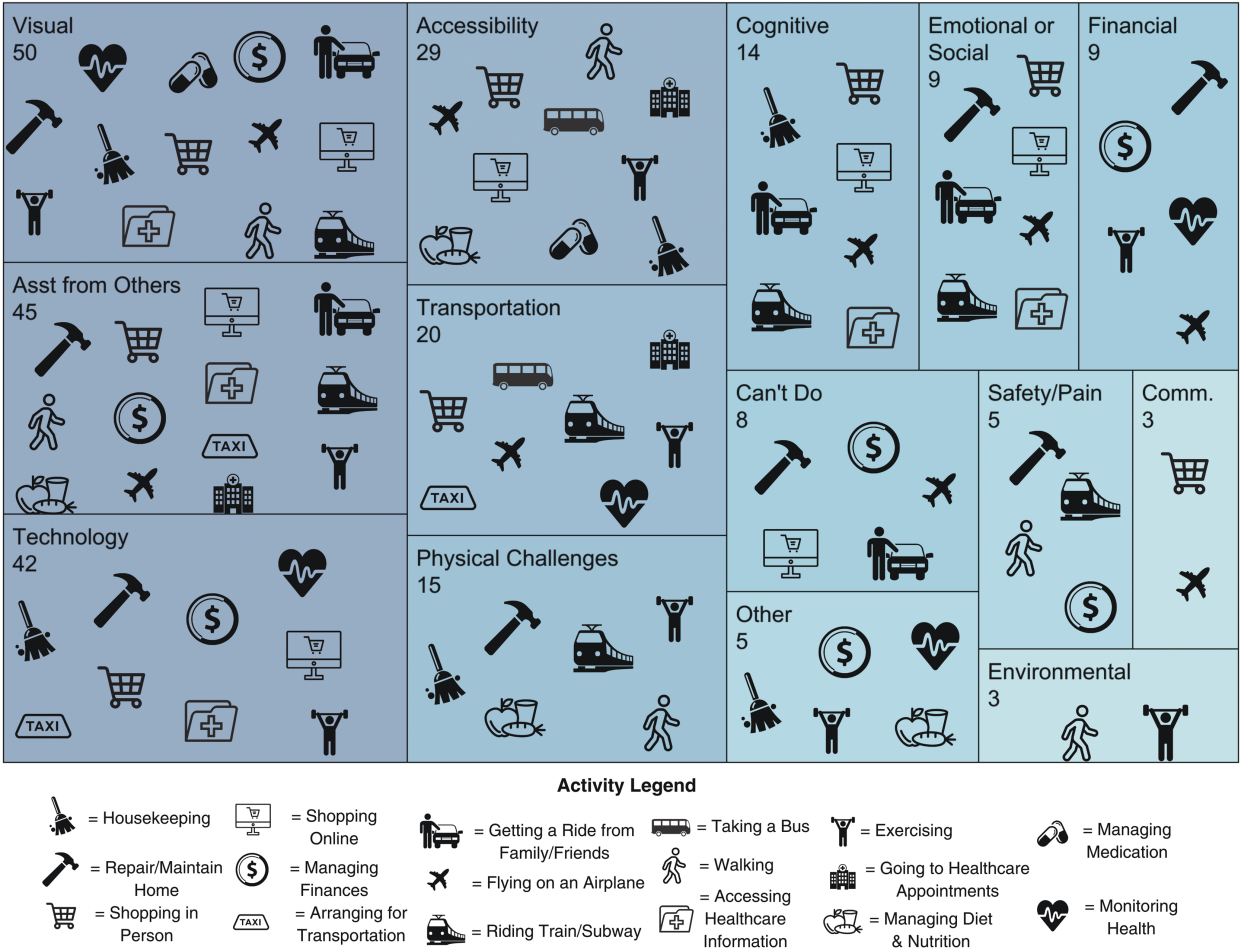
Treemap displaying proportion of challenge codes (*n* = 257) reported across 17 activities. Data are presented in nested rectangles that each represent distinct challenge codes, proportional in size to the number of times the challenge was reported across activities. Within each rectangle (1) the frequency of each challenge type is listed below the name of each challenge and (2) the presence of an activity icon indicates the challenge type was reported for that activity (challenge codes reported fewer than three times excluded from figure).

Figure 2 provides a treemap visualization of the data, which highlights recurrent challenges across activities. For example, financial challenges were mentioned nine times across five activities, including: repairing/maintaining home, managing finances, monitoring health, exercising, and flying on an airplane. The five most frequently reported challenges included: visual, need for assistance from others, technology, accessibility, and transportation. The treemap also reveals the types of challenges reported for a specific activity. For example, with exercising (weightlifting icon), nine different challenges were reported, including: visual, need for assistance from others, technology, accessibility, transportation, physical, financial, other, and environmental. In contrast, managing medication (pill icon) had only two different types of challenges reported (i.e., visual, accessibility).

To provide context on the lived experience of these activity challenges, Tables 3–6 highlight challenges for specific activities along with illustrative, example quotes from participants across the four categories.

#### Household tasks

Table 3 shows Household tasks. For repairing and maintaining the home, many reported challenges shared the common theme of outsourcing tasks that are difficult, time-consuming, or potentially unsafe. Participants described concerns about the cost of services and trusting service providers. Some participants reported no longer doing certain desired home improvement activities, such as gardening. For housekeeping, challenges were primarily related to the inability to see things that need to be cleaned up (e.g., messes, stains) and lacking spatial awareness for tasks, such as avoiding obstacles while vacuuming.

**Table 3.** Household Tasks Category: Example Challenges and Quotes

Activity	Challenge code and participant quote
Repairing and maintaining home	*Assistance from others:* “Getting somebody that I can trust to do the job is the biggest problem.” *Cognitive or knowledge limitation:* “There are safety issues with repairs. The tools that are required require vision.” *Emotional or social:* “I probably could do these things, but it just is so time consuming, so it would be frustrating for me.” *Safety/pain:* “Well the gardening and then cutting the grass, I wouldn’t even try to do that because I wouldn’t want to get hurt, and then changing light bulbs, I ask my aid to do that because I had tried to pull the lightbulb out of the lamp and I cut my hand earlier this year.”
Housekeeping	*Visual limitation*: “Because I can’t see, not knowing, I think things are clean sometimes. It may visually look dirty to you, but it feels clean to me … If the grandkid spills something on the floor, I go to wipe it up it and it feels clean to me. Then my wife comes, ‘there’s still a big spot there.’”

#### Shopping and finances

Table 4 depicts Shopping and finances. For managing finances, several participants reported not being able to independently complete tasks, such as banking and signing receipts. This reliance on others to engage in tasks was primarily due to vision limitations, specifically with reading, but some attributed their need for assistance to physical limitations (e.g., carpal tunnel) or limited technology familiarity.

**Table 4.** Shopping and Finances Category: Example Challenges and Quotes

Activity	Challenge code and participant quote
Managing finances	*Assistance from others*: “The print that is immediately available by their scanners or by their receipts are difficult to read with my eyesight as it is, and so I have to rely on others to read to ensure that I understand what I’m purchasing.” *Physical:* “It’s just being able to interact with [a computer] because of my carpal tunnel. As far as me being independent at doing financial transactions, I do very few.” *Can’t do or don’t do the task:* “When I go to restaurants now, I basically stopped trying to fill out the credit card receipt. I have my friends fill them out. I could use my magnifier to read what the amount is, but then to add the tip, and find the line to add the tip. I’m left-handed and I hook my hand when I’m writing, which casts a shadow when I use a magnifier so that can be a real problem.”
Going shopping in-person	*Visual limitation*: “As a totally blind person, I’m not able to simply go in and find what I want. I can use a barcode reader, and there are other phone apps that do similar types of things, but if I’m going to try to read every barcode … I’m going to be in a supermarket for hours and hours.” *Emotional or social:* “I used to get upset … I go in [the store] and I start reading the labels with my magnifier, and I realized the way that I be doing things, it’s the same way that somebody would be doing who would be stealing. I used to work at a center for visually impaired people and we used to tell people ‘don’t be alarmed when you be shopping alone’, because you might be looking suspicious. Your face be all down in the clothes like you might be trying to hide something under your arm … if a person doesn’t know what it is you’re dealing with, they very well [could think] you are stealing.” *Communication:* “Things are really hard to describe. [There are] basic primary colors, but now you have seafoam green and teal, when somebody tries to describe those colors to me, they’re not in my memory bank.”
Shopping online	*Technology, tools, or devices:* “I have magnification on my computer, [but] very little at one time fits on the screen, and so trying to maneuver through the pages to see what’s there is very difficult for me. There have been a couple of occasions where I double ordered something.” *Accessibility:* “Websites that deal a lot with pictures of what they’re selling and not descriptions of what they’re selling [is challenging].” *Cognitive or knowledge limitation:* “I really don’t know how to do it. I just got an iPhone and I’m trying to learn how to use it, I would like to be more able to do online shopping. I would like to know everything that would help me to make me more independent like I used to be.”

Distinct challenges were reported for in-person shopping compared to online shopping. Many in-person shopping challenges were attributed to visual limitations, such as identifying items and reading labels. Participants described using AT, such as magnifiers and barcode readers, to shop in-person, yet these devices were reported as being tedious and time-consuming to use, especially when shopping for numerous items. Shopping for clothes was described by some as particularly difficult, given the wide array of colors, sizes, and styles. Participants expressed challenges in obtaining helpful descriptive information about the items from informational tags as well as other people. One individual shared their experience of being perceived as being suspected of stealing by store employees when using their strategies for clothes shopping, such as using a magnifier and holding items closely.

Online shopping challenges were related to the use and accessibility of technology. For some, the challenge was simply not knowing how to use a computer or smart phone for shopping. Among participants who were familiar with online shopping, website accessibility was a common issue. Several participants described being unable to use certain shopping websites because the content was not “screen-reader friendly.” Screen readers are designed to read aloud the text on a screen, yet participants reported that many shopping websites were not compatible with their screen reading software. Example issues include websites not providing image descriptions (i.e., alternative text) or appropriate headers to support content navigation. One participant, who used computer screen magnification, described how it was difficult to navigate the online shopping process, from browsing to checkout, because the text on their screen was so large that relevant information on the website could not be seen.

#### Transportation

Challenges were reported for all modes of transportation we evaluated (Table 5). For flight travel, participants reported difficulty navigating the airport. One participant described how it is often the standard protocol of airport staff to put customers who need assistance in a wheelchair and push them, although it was noted that most would prefer someone to walk with them as a sighted guide. Challenges with walking were primarily related to navigation. Participants described safety concerns, namely tripping and falling, especially when walking on uneven sidewalks or landscapes. Some individuals discussed challenges using wayfinding technologies, such as smartphone maps and apps. One participant who used a wayfinding app designed for individuals with visual impairment found that, although the app read aloud helpful information, it often gave too much detail, which was distracting and inhibited other critical observations (e.g., auditory crosswalk signals). Physical challenges associated with walking as a pedestrian (e.g., fatigue, limited stamina) were also reported.

**Table 5.** Transportation Category: Example Challenges and Quotes

Activity	Challenge code and participant quote
Flying on an airplane	*Communication*: “In order to get assistance at the airport as a blind person, often times they want you to ask for a wheelchair. But for a blind person, they really don’t want to get in a wheelchair. They want a sighted guide to help walk them. That negotiation is really difficult. Those who do fly alone and want assistance, they resign themselves to sitting in the wheelchair and being pushed.”
Walking	*Technology, tools, or devices:* “Some of the wayfinding apps that are designed for people who are blind work really well, but on the other hand, I also think sometimes they give you too much detail. Trying to listen to something in your ear, whether it’s with the Bluetooth device or you’re holding it to your ear is distracting. You’re not focused, it takes away from the focus of listening to the sounds around you, whether you’re walking, using echo location, or most people use a cane or a dog. It takes away from being able to pay attention to what’s around you.” *Physical:* “I’m just not fit to do that, you know. I just have fatigue, not stamina.” *Safety/Pain:* “I am worried about tripping, falling. I have to use a walking stick.” *Environmental:* “Unlevel sidewalk, unlevel transitioning sidewalks, and transitioning landscapes. I have no depth perception so it’s very difficult and having a guide dog, [or] using a cane when I’m not using the guide dog, helps tremendously.”
Getting a ride from family or friends	*Assistance from others:* “When I ask them to do it, they can’t do it. They might not be available. They might not have the time to come pick me up.” *Emotional or social:* “The emotional aspect of asking for somebody feels very vulnerable. [I’m] not always comfortable with that.”
Riding a train or subway	*Visual limitation:* “Getting on the train or the subway is not a real challenge but locating a seat that’s empty, knowing your stops and how to get out of there. I wouldn’t do subway or a train unless I’ve done that route with a sighted person before.” *Cognitive or knowledge limitation:* “I really don’t know the system, I don’t know some of the places where you catch the subway. I don’t feel very safe. There are no two of the same of the subway stations. I just don’t know very much how to do it and I don’t have a lot of resources to change that.” *Emotional or social:* “The fear of falling into the tracks, and the crowds. People tend to push. I almost got pushed down the escalator the last time I was in one of the train stations, and I decided I’m never gonna use a train again.”
Taking a bus	*Safety/pain*: “Getting on and getting off the bus, and having to stand up, or try and sit when the vehicle is moving. I try to keep my both hands free, if I can, because I’m afraid I’ll fall otherwise.”
Arranging for transportation via taxi, Uber, or Lyft	*Technology, tools, or devices:* “I don’t know how to use the technology to use Uber and Lyft.” *Transportation:* “I use my Uber app on my phone. The challenge is, when I need one, there’s never one in there.” *Safety/pain*: “Trusting the person that’s a total stranger to get into their vehicle.”

Challenges with getting a ride from family or friends were related to the availability and reliability of others. Some participants expressed emotional challenges with getting a ride, such as feeling burdensome and vulnerable. For riding a train or subway, challenges included vision limitations, lack of knowledge about the system, and unfamiliar environments. One participant described their fear of falling onto the tracks, whereas others described negative experiences navigating crowds on trains and subway systems. Safety concerns about falling were reported for using the bus, especially when getting on and off, and preparing for stops when the bus is in motion. For taxi and ride-share services, participants reported issues related to not knowing how to use the technology and limited availability of drivers. Additionally, participants shared concerns about having to trust a stranger to drive them around because they cannot see what the driver looks like or what route they are taking.

#### Managing health

Table 6 highlights challenges for Managing Health. Accessibility was the most reported challenge for exercising. Participants described how many exercise machines (e.g., treadmills) are not accessible to them because they often feature touchscreen interfaces without tactile buttons, and only provide visual, rather than audio, feedback. Group exercise classes were also described as inaccessible, as many instructors rely on visually demonstrating movements without adequately describing the movement (e.g., direction, specific body parts engaged). Other exercise challenges included transportation to fitness centers and personal limitations (e.g., physical declines, lack of motivation).

**Table 6.** Managing Health Category: Example Challenges and Quotes

Activity	Challenge code and participant quote
Exercising	*Accessibility:* “[At the gym] all of their machines are like electronic and digital. So if I get over there, I still can’t operate them. Even the treadmill, it’s digital, so nonaccessible to people who are blind or have low vision. I tried to go to a water aerobics class, and the instructor, is up there like ‘do this!’ and if you can’t see, or can’t see well, you have no idea what they’re doing.” *Physical:* “Just being able to do the whole routine. Let’s say the exercises in the class that I’m in, I will try them, and at one time, that would have been no problem for me. So, coming to terms to realize that I can do maybe eighty-five to ninety percent of them, but the rest I have to let go..” *Transportation:* “I would like to go to a gym and participate in some type of exercise. But because of transportation—because of income—I am not really able to do that.” *Other challenge:* “There is a gym in the next town that is specifically designed for disabled people. I really should go there and get involved in an exercise program, but it takes so much time to stick with it for 3 or 4 times a week, and it takes time away from other things.”
Managing diet and nutrition	*Technology, tools, or devices: “*I have a problem with reading the back of the box, like directions. [My reader] usually won’t read the back of a box, because of the fonts.” *Cognitive or knowledge limitation*: “The reading of food labels, understanding what to eat and what not to eat beyond basic things. I don’t understand like, for examples, carbs and all that kind of stuff. “
Accessing health information	*Technology, tools, or devices*: “They always want you to do it on the Internet, almost without exception. And that means I have to get somebody who has the patience to sit down and go through it with me. If you try to call any company you inevitably are faced with phone service, but none of these organizations keep people to keep up with the telephone anymore, so you inevitably end up waiting a long time. If you finally get somebody to talk to, [they] start wanting to know all kinds of answers to questions, and if you haven’t prepared yourself beforehand, you may be faced with hanging up and gathering up more information and calling back and going through it again.” *Accessibility:* “The challenge is the website, because the website is not user friendly for screen readers.”
Monitoring health	*Technology, tools, or devices: “*Blood glucose meters. They have made so much improvement and that glucose monitor can work by bluetooth into your cell phone and everything, [but] they have not taken in any consideration to make this [usable] for the visually impaired. Showing a picture of a scale is not going to help us.” *Transportation:* “It’s a huge problem, transportation. Instead of everything being within a few blocks, it’s all over the place. One’s on one side of town, the other one’s the other side of town, and you can’t walk from one to the other.” *Financial:* “I can get to the doctor, I can listen to what they say, but you know, financially, I can’t eat like they want me to eat because fresh vegetables and fruits are not cheap, and between paying mortgage, and utilities and getting back and forth to work, and paying people to assist me in this matter or that matter, it doesn’t leave too much to help me with my health problems.” *Safety/pain*: “I don’t cook in the residence I live in because I’m not comfortable - there’s no vent for the stove. There’s no window I can open to let smoke out when I burn something, or the doorways don’t have screens so I can’t open that. I don’t cook as often as I should.”
Managing medication	*Visual limitation:* “Making sure I’m taking the right medicine and the right dosages since I can’t read the label. I know that there are companies that provide audible labels that will verbally tell you what’s in the container, how many milligrams, and how frequently you’re supposed to take it, but mine doesn’t. I have to scan them with my scanner to read what they are. What I usually do is set them up for a 7-day supply in the little pill boxes.” *Cognitive or knowledge limitation*: “Taking it, trying not to forget.”
Going to healthcare appointments	*Assistance from others:* “Getting in and out of the building and moving around in the doctor’s office, you have to rely on someone. Some offices may have a security or someone that will help you up to your floor or suite that you’re going to, and some of them don’t.” *Transportation:* “Getting transportation. Other than that, no problem.”

For managing diet and nutrition, participants discussed challenges in reading information, such as food labels or recipes. Many reported having issues with assistive devices that were intended to support these activities (e.g., handheld readers unable to read contents on the back of a product due to the font size). For managing and monitoring health, some participants mentioned struggling with making healthy meals at home due to the high cost of fresh food, as well as safety concerns using the stove. Participants described barriers to accessing healthcare information, as processes have shifted to being mostly online with limited opportunities to speak with a representative for support. Some noted that their healthcare and insurance provider websites were not screen reader accessible. One participant described their difficulty keeping track of online portals, account information, and passwords.

Transportation challenges getting to/from healthcare appointments and the pharmacy were discussed. Participants described how healthcare monitoring devices are often not accessible to people with vision impairment. For example, two individuals described issues with talking blood glucose monitors, devices intended to help users with vision impairment manage diabetes; they reported trouble lining up the test strip with the blood sample (due to visual challenges) and the phone application for the meter only providing visual infographics (e.g., photos of scales) instead of text information that could be read aloud by a screen reader.

Regarding medication, participants described challenges reading labels on prescriptions and over-the-counter medicines to ensure they are taken at the correct dosage and time. Issues remembering to take medications were also reported. For going to healthcare appointments, challenges were associated with having to rely on other people, both for transportation and navigating healthcare facilities.

## Discussion

This qualitative study explored challenges with a broad range of IADLs in the home and community among people aging with vision impairment. Participants discussed a wide range of IADL challenges that are most difficult for them personally. Across participants, the most difficult, and therefore most frequently discussed, activities were repairing and maintaining the home (Household Tasks), managing finances (Shopping and Finances), flying on an airplane (Transportation), and exercising (Managing Health). The details they described highlighted many themes but most frequently the nature of the challenges related to visual limitations, need for assistance from others, technology issues, lack of accessibility, and transportation barriers. These data provide insights for technology design and innovation to support activity participation for this population.

### Visual Challenges

Not surprisingly, challenges explicitly related to visual ability were the most frequently reported type of IADL challenge and included reading fine print (e.g., medication labels), distance vision (e.g., in-person shopping, identifying messes or spills), and outdoor mobility (e.g., navigating public transportation systems). These findings are consistent with prior research (Cimarolli et al., 2012; Lamoureux et al., 2004). Previous findings have shown that poorer visual ability (in terms of acuity, contrast sensitivity, and useful field of view) is independently related to longer times for completing certain IADL tasks among older adults (Owsley et al., 2001). We found that people aging with vision impairment reportedly experience a broad array of challenges with IADLs, that go beyond visual limitations, including both personal factors (e.g., physical, cognitive, emotional) and environmental factors (e.g., accessibility, financial, transportation). Design of supportive solutions for people aging with vision impairment must consider that users may experience a combination of challenges that can affect their ability to engage in an activity and/or effectively utilize a service or device.

### Relying on Assistance From Others

Participants frequently reported struggles associated with getting or requiring assistance from others for IADLs. Key issues included: being dependent on others to complete tasks and challenges with hiring help (e.g., cost, trust, reliability). Our findings suggest people aging with vision impairment want to perform activities as independently as possible, consistent with prior research that identified loss of independence as the most challenging adjustment to vision impairment (Nyman et al., 2012). People aging with vision impairment are accustomed to using their voice to support everyday tasks and could benefit greatly from the growing market of voice-controlled technologies (Bhowmick & Hazarike, 2017). Voice-activated digital assistants (e.g., Alexa, Siri, Google Home) have tremendous potential to support independence in IADLs, from making and reading grocery lists, to delivering medication reminders, to controlling various smart home devices (e.g., smart light bulbs and vacuums). However, for these devices and skills to be effective, they must be intuitive to set up, learn, and use, and must be designed to accommodate users with diverse abilities (Kadylak et al., 2022).

### Transportation Challenges

Transportation was reportedly a challenging aspect of activities outside the home and a challenging IADL in and of itself. This largely nondriving population could benefit greatly from accessible public and private transportation. It cannot be assumed that they can rely on rides from family or friends, as this option may not be available, convenient, or desired. Participants’ desire to use public transportation was coupled with a need for navigational technology that facilitates spatial awareness, safety, and ease in using these systems. Mobile apps that offer real-time transit information and step-by-step wayfinding information in an accessible format hold great promise to enhance independent community mobility for this population. Insights from participants suggest the need for customizable transportation and wayfinding apps that enable users to pick and choose what and how information is relayed. For example, we found that if pedestrian wayfinding apps provide too much auditory information, they can create cognitive overload for people with vision impairment, as they also rely on observing sound cues in their environment. These results also identify an opportunity for app-based ridesharing services, like Uber and Lyft, to increase safety, trust, and confidence for users with vision impairment by providing accessible information that confirms their driver and route progress.

### Technology and Accessibility Challenges

Many technology challenges were interconnected with accessibility issues (e.g., inaccessible devices, applications, and websites), so these key challenges are discussed collectively here. Insights from people aging with vision impairment demonstrated how the internet can be involved in supporting all IADLs, either directly (e.g., online banking) or indirectly (e.g., a website for finding home repair professionals). Our findings suggest that website accessibility, or lack thereof, is a central issue facing people aging with vision impairment. There are known solutions to make online content accessible for users who are visually impaired, such as minimum contrast standards, text resizing capabilities, and screen reader-accessible content (e.g., describing images using alternative text; using headers to aid in page and website navigation). The Web Content Accessibility Guidelines are generally accepted as the accessibility standards for webpages and mobile applications and provide instructions for website and app owners to ensure that their platforms are accessible to, and do not discriminate against, users with varying disabilities (W3C Web Accessibility Initiative, 2022). Recently, the Americans with Disabilities Act (ADA) specified that websites and mobile applications are considered “places of public accommodation,” and therefore, state and local governments as well as businesses open to the public must provide accessible web content (ADA.gov, 2022). However, there is clearly a gap between this requirement and current practice. In fact, an increasing number of lawsuits confirm the requirement of public businesses to provide accessible web content (Palmer & Palmer, 2018).

Despite reported challenges with web accessibility, most participants described using personal computing devices (e.g., laptops, smart phones, and tablets) to carryout IADLs, from shopping, to banking, to accessing healthcare information. For these personal devices, participants mentioned using a wide variety of applications and software designed for users with vision impairment (e.g., bar code scanners, color identifiers, screen readers) as well as built-in accessibility features (e.g., magnification, large text). These devices hold great potential to support this population, as they offer the benefit of an integrated platform that is mainstream, unobtrusive, and lacks the stigma of separate AT (Hakobyan et al., 2013). Moreover, digital interfaces are dynamic and adaptable for users with a wide range of abilities, which can facilitate the implementation of universal design principles (Sanford & Remillard, 2021). In line with prior research, some reported technology challenges were related to a lack of structured, accessible training oriented toward people with vision impairment, which can play an important role in technology adoption (Piper et al., 2017; Tapu et al., 2020).

This study identified a few key opportunities for technology innovation that are activity-specific. First, given the extensive challenges reported for in-person shopping, there is a critical need to make online shopping accessible for people aging with vision impairment so they can obtain everyday goods, from groceries to clothing, from the convenience of home. To do so, online retailers must provide comprehensive product information on their websites in an accessible format so visually impaired users can make informed purchases.

With regard to healthcare, our findings suggest that, in addition to making healthcare communication and portals accessible, providers should maintain some capacity for phone support to be inclusive of individuals who are less familiar with the internet, or unable to navigate the website due to accessibility issues. Participants described instances where healthcare devices and applications did not provide feedback that was useful and usable, indicating the need for devices to be designed with intuitive cueing and facilitate comprehensive audio description. For exercising, participants described barriers to engaging in in-person classes and utilizing fitness facilities (e.g., transportation access, inaccessible classes, and equipment). Results indicate the need for technology-based exercise equipment to be multimodal (e.g., audio input/output, tactile buttons), as touchscreen interfaces alone are not inclusive of users with vision impairment. Exercise programs delivered remotely via tele-technologies like videoconferencing (e.g., Zoom) can be especially impactful for this population as they offer the convenience of participating from home and utilize the user’s personal computer or smart phone, which they can adjust to their accessibility needs and preferences. Moreover, these telewellness programs can expand access to accessible exercise programs that provide sufficient verbal cueing and instructors who are sensitive to the needs of people with vision impairment (Mitzner et al., 2022).

### Limitations

There are a few limitations of this study to note. The convenience sample was majority White/Caucasian with high education and high self-perceptions of health. Activity challenges might be greater among racial/ethnic minority populations and those with lower education due to known disparities in access to transportation, healthcare, and technology (Kaye et al., 2022; Meade et al., 2015). Causes of vision impairment were not strategically sampled, but represent a variety of conditions, diseases, injuries, and abnormalities that were congenital or acquired at an early age. Nevertheless, the diversity of the sample in terms of degree, functional limitations, and cause of vision impairment is a strength of this study, as the population aging with vision impairment is diverse and is likely to have unique experiences that are important to consider to develop effective solutions (Bhowmick & Hazarike, 2017).

The interviews were structured to elicit conversations from participants regarding their *most difficult* activity(ies) in each category. That is not to say they did not experience difficulties with other activities that were not the focus of the discussion. The current paper focused on challenges with IADLs and not participants’ strategies/solutions for managing activity challenges, which are captured in the ACCESS data set. To effectively support activity performance of people aging with vision impairment with technology interventions, there is a need to understand more about what works, what does not, and if/how new emerging technology is being adopted and used long-term. Such knowledge is necessary to inform the design of effective products and services for this understudied population and is an important area of future exploration.

### Implications

Our findings highlight many opportunities for design and technology innovation to support activity participation and independence among older adults with vision impairment. Two promising types of technologies to support IADL performance for the challenges noted include (1) voice-activated digital assistants and (2) apps for smart phones and personal computing devices, which have digital interfaces that are adaptable for users with a wide range of abilities. There is a clear need to make existing technologies accessible for people with vision impairment with the most pressing issue being websites that are not compatible with screen readers. Our findings emphasize the need for broader mandates and policies that both enforce and incentivize the development and maintenance of accessible web content. There is also a need to make nontechnology items (e.g., price tags, food, and clothing labels) more accessible to AT devices, such as scanners. Given that technology has become ubiquitous in everyday life (e.g., ATMs, shopping, healthcare portals, rideshare apps), technology training for these everyday technologies that is targeted at people with vision impairment is necessary. Vision rehabilitation centers, which offer a variety of resources to help individuals with vision impairment engage in IADLs (e.g., community mobility training, AT matching, and training), are ideal for delivering such training but require dedicated, sufficient funding to offer these services on an ongoing basis.

## Supplementary Material

gnad169_suppl_Supplementary_Material

## Data Availability

Data are not publicly available. Researchers interested in analyzing the ACCESS data set can contact the study team to explore opportunities for archival analyses in partnership with the research team. Study methods, assessments, and the coding scheme are detailed in technical reports available from the authors (Remillard et al., 2018). Preregistration is not applicable given the nature of the study.
