# Supplementary material for: Everyday Challenges for Individuals Aging With Vision Impairment: Technology Implications
Source: Gerontologist. 2023 Dec 20;64(6):gnad169. doi: 10.1093/geront/gnad169 (PMC11102008; doi:10.1093/geront/gnad169)
Supplement: gnad169_suppl_Supplementary_Material [file gnad169_suppl_supplementary_material.docx]

**Online Supplementary Material**

**Supplementary Table 1**

*Coding Scheme for Challenges for the ACCESS Vision Group*

| Challenges Codes & Subcodes | Definition | Example Quotes |
| --- | --- | --- |
| Accessibility | Related to barriers that limit the use and access of people with disabilities. “Accessibility is when the needs of people with disabilities are specifically considered, and products, services, and facilities are built or modified so that they can be used by people of all abilities”. (CDC, 2017; Yanchulis, 2002). |  |
| Access to assistive technology/tools | The system/entity lack access or availability to assistive tools/technology such as closed captioning, hearing aids, mobility aids | *“Most of the movie theaters around where we live don’t have any kind of descriptive videos, so I know in some of the larger cities, they offer some kind of a headset that will describe the activities on the screen, but we don’t have that and so I just sit there."* |
| Access to information | The system/entity lacks tools to assist users in identifying necessary information such as audible messages or screen-reader accessible text. | *“Making sure I'm taking the right medicine and the right dosages and everything since I can’t read the label and everything. I know that there are companies that provide audible labels that will verbally tell you what's in the container, how many milligrams, and how frequently you're supposed to take it, but mine doesn't.”* |
| Physical access | Access to buildings, private or public spaces, the living/built" environment, and any other place a person might need or want to go for work, play, education, etc. Physical access includes accessible routes, curb ramps, parking and passenger loading zones etc. And includes access to things that are physically out of reach (too high, too far). | *“Getting to where I need to be...The room numbers, where to sit, etc.”* |
| General | A challenge related to general accessibility, nothing specific. | *“Shopping online is difficult because a lot of the time it’s not accessible to blind people, or people that have a loss of vision.”* |
| Other | Other forms of specific accessibility challenges that do not fit within other subcodes. | *“I guess the most challenging part is the airline's readiness to do with having a guide dog on the airplane. I always let them know in advance when I make my reservations that I'll be traveling with a guide dog and I'd prefer to sit up at the bulk-head where there's the most amount of floor space for my dog to lay down and so that the dog will be comfortable. The dog travels and flies very well whether it's on a plane, on a train, or a bus, he's very well trained, he's never an issue. It's more the handlers that transport us that have issues about it because either they don't have those skills or the background knowledge to know what the laws are or how to deal with that situation.”* |
| Technology/Tools/Devices |  |  |
| Ease of use / Complexity | The technology is difficult to use and not intuitive. Complexity of the tool/technology takes a lot of effort to use, causing inconvenience. | *“The challenge is the website, because the website, for example, for my family medicine center, is not user friendly for screen readers.”* |
| Features | Related to service/maintenance.  Specific features of the technology are challenging  Speed of the technology (fast or slow)/Durability of the device or technology/poor content or poor quality of device/appearance of the technology or device/poor fit or size of the technology or device  Too many or too few features or programming options. | *“A lot of times...a handheld magnification device may not be strong enough and it may very well be that I'm going to need use a computer and some companies or whatever don’t like using or load certain kind of software into their systems.”*  *“Generally, the color combinations that manufacturers choose for their display screens…I would do better with a very high-contrast black background, or yellow or green display.”* |
| Reliability | How well a technology works. | *“I could fill out a form using a combination of the scanner and a video magnifier. I mean the scanner would be doing most of the reading and I would sort of kind of follow around and see the blank lines, and find the blank lines to sign…I mean it isn’t always really precise. So, it’s an issue.”* |
| General | Non-specific challenge related to the use of tools/technology/devices. | *“Anything that requires tools and being able to make the repair.”* |
| Other | Other specific challenges about the tools/technology that do not fit within other subcodes. | *“It’s using websites with the technology that I have, and taking full advantage of what a retailor offers online...is pretty inaccessible.”* |
| Assistance from Others | Getting/needing assistance from others is the source of the challenge/problem.  Family/Friends/etc. are not available to help, or it is inconvenient for family/friends/etc. to help.  Family/Friends/etc. do not do a sufficient job when helping.  Cannot do the task without assistance. | *“Going out on movies, or things like that, you have to depend on someone to guide you.”*  *“...some stores that I ask for assistance, it’s a long wait.”*  *“When I ask them to do it, they can’t do it.”* |
| Can’t Do/Don’t Do the Task | Can’t or don’t do the task but no specific reason provided. | *“When I go to restaurants now, I basically stopped trying to fill out the credit card receipt.”* |
| Financial | The cost of something is a challenge (could be the cost of a technology/tool or the cost of assistance).  Too expensive or cannot afford. Limited to services/resources covered by insurance. | *“I can get to the doctor, you know, I can listen to what they say, but you know, financially, I can’t eat like they want me to eat...”* |
| Transportation |  |  |
| Not accessible | Transportation lacks features that make it usable for people with disabilities. | *“Getting to the point you need to be [at the airport] …Assistance in getting to where I need to be.”* |
| Not available | Transportation may be unavailable or at inconvenient distances or “I have to rely on the availability of parking.” locations (CDC, n.d.). | *"I use my Uber app on my phone...The challenge is, when I need one, there’s never one in there...I go another day, or go later...Just keep checking."* |
| General | Non-specific challenge related to the use of transportation. | *“Transportation.”* |
| Other | Other specific challenges about transportation that do not fit within other subcodes. | *“Getting on and getting off the bus, and having to stand up, or try and sit when the vehicle is moving.”* |
| Safety / Pain | Related to safety of self or others or pain to self or others.  Physical discomfort / Injury to oneself or others. | *“The fear of falling into the tracks, and the crowds. People tend to push.”* |
| Cognitive / Knowledge Limitations | Not knowing how to do something, unfamiliar with the task. Task being difficult because of memory failure of memory issues (including procedural and declarative knowledge issues; Anderson, 1983). | *“I really don’t know the system...”* |
| Communication | Related to difficulty conveying information to, and understanding information from, others. | *“...it can be difficult to hear people in subways to ask for directions...”* |
| Emotional / Social | Related to emotional difficulty, or some sort of social challenge.  Impatience, frustration, embarrassment. Negative attitudes/behaviors towards someone's disability (CDC, n.d.). | *"The emotional aspect of asking for somebody feels very vulnerable…[I'm] not always comfortable with that."* |
| Environmental | Outdoor, environmental variables such as weather, climate, other and natural aspects. | *"Street crossing at some places...the lights aren’t there and there’s no audio signal and the traffic is going and coming.”* |
| Transferring | The act of transferring: in and out of a shower, on and off the toilet, in and out of bed is the source of the challenge. | *“I guess, maybe getting off the toilet. I have grab bars.”* |
| Physical |  |  |
| Physical Strength / Endurance Limitations | Related to lack of strength; objects/tools/body weight too heavy/participant not strong enough to complete task, or struggle with task because of a lack of physical strength (upper/lower body strength). Source of challenge may also be exhaustion, fatigue, lack of energy or lack of endurance. | *“Getting up or, or standing for long periods of time.”* |
| General Health / Physical Limitations | Constraints caused by health of physical problems that were not specific to motor, visual, or auditory limitations. Common themes: health problems effectively shut people out of engaging in activities such as housekeeping, cooking, and traveling (Rogers et al., 1998). | *“It's just having access to a computer and being able to interact with it because of my carpal tunnel.”*  *“Well, I think my sense of balance isn't as good as it used to be.”* |
| Mobility Limitations | Mobility challenges or mobility abilities/limitations including gross motor movement, fine motor movement balance (Rogers et al., 1998). | *“Just walking, my legs are not very supporting so I have difficult with that with walking long distances.”* |
| Visual Limitation | Related to one’s abilities/limitations with seeing that result in difficulties performing activities. | *"Because I can’t see, not knowing, I think things are clean sometimes. It may visually look dirty to you, but it feels clean to me.”* |
| Auditory Limitation | Related to one’s abilities/limitations with hearing that result in difficulties performing activities. | *“The most important thing would be if there were an emergency situation and I couldn't hear the smoke detector or I couldn't hear someone at the door trying to warn me that the house was on fire or that something was going on.”* |
| Other Challenge | Challenge that does not fit within any of the other challenge codes. | *“Standing in line and waiting to pay, standing in line, long wait, long line. I'd rather do it [pay bills] by mail.”* |

*Note*. Only codes applied/relevant for the vision group are included.
